# A longitudinal study of facial asymmetry in a normal birth cohort up to 6 years of age and the predisposing factors

**DOI:** 10.1093/ejo/cjad012

**Published:** 2023-04-10

**Authors:** Anniina M Launonen, Ville Vuollo, Henri Aarnivala, Tuomo Heikkinen, Pertti Pirttiniemi, A Marita Valkama, Virpi Harila

**Affiliations:** Department of Oral Development and Orthodontics, Oulu University Hospital, Oulu, Finland; Department of Oral Development and Orthodontics, Unit of Oral Health Sciences, Faculty of Medicine, University of Oulu, Oulu, Finland; Medical Research Center Oulu, Oulu, Finland; Department of Oral Development and Orthodontics, Oulu University Hospital, Oulu, Finland; Department of Oral Development and Orthodontics, Unit of Oral Health Sciences, Faculty of Medicine, University of Oulu, Oulu, Finland; Medical Research Center Oulu, Oulu, Finland; Department of Children and Adolescents, Oulu University Hospital, Oulu, Finland; PEDEGO Research Group, Oulu, Finland; Department of Oral Development and Orthodontics, Oulu University Hospital, Oulu, Finland; Department of Oral Development and Orthodontics, Unit of Oral Health Sciences, Faculty of Medicine, University of Oulu, Oulu, Finland; Medical Research Center Oulu, Oulu, Finland; Department of Oral Development and Orthodontics, Oulu University Hospital, Oulu, Finland; Department of Oral Development and Orthodontics, Unit of Oral Health Sciences, Faculty of Medicine, University of Oulu, Oulu, Finland; Medical Research Center Oulu, Oulu, Finland; Medical Research Center Oulu, Oulu, Finland; Department of Children and Adolescents, Oulu University Hospital, Oulu, Finland; PEDEGO Research Group, Oulu, Finland; Department of Oral Development and Orthodontics, Oulu University Hospital, Oulu, Finland; Department of Oral Development and Orthodontics, Unit of Oral Health Sciences, Faculty of Medicine, University of Oulu, Oulu, Finland; Medical Research Center Oulu, Oulu, Finland

## Abstract

**Objectives:**

This prospective, population-based cohort study aimed to investigate the development of facial asymmetry up to 6 years of age using a three-dimensional (3D) soft tissue imaging method in a normal population. In addition, the study sought to identify potential predisposing factors to facial asymmetry.

**Methods:**

A total of 102 newborns were enrolled in the study at birth. 3D stereophotogrammetric images of the head and face were analysed at the ages of 12 months (T1), 3 years (T2), and 6 years (T3). The surface-based analysis involved the calculation of the average distance (mm) and the symmetry percentage (%) between the original and mirrored surfaces. For landmark-based analysis, the distance of facial landmarks to the facial midline was examined.

**Results:**

The final analysis included 70 (68.6%) subjects. Surface-based analysis showed a significant improvement of facial symmetry from T1 to T3 in all facial areas. Landmark-based analysis showed that upper facial landmarks were located, on average, slightly on the left and lower facial landmarks slightly on the right in relation to the facial midline (*P* < 0.001).

**Limitations:**

The size of the study population was limited. Facial posture may affect the reliability of the results, especially in younger children.

**Conclusion:**

Facial asymmetry is detectable in early childhood and tends to reduce with age in young children. The lower face deviates slightly to the right, and the upper face to the left in relation to the facial midline. Possible predisposing factors for facial asymmetry at the age of 6 years include deformational plagiocephaly, sleeping position, and previous facial asymmetry.

## Introduction

Facial asymmetry is a subject of interest in a large number of studies. The role of environmental factors during development has long been investigated. Furthermore, recent studies have identified a stronger role of genes and heredity in the development of asymmetry (1). Facial asymmetry detected in childhood may be caused by prenatally occurring anomalies, deformations, and disturbances. Also, postnatally occurring unilateral growth disturbances, traumas, infections, or pathological conditions may cause slowly evolving facial asymmetry. In addition, multiple functional or developmental factors, such as habitual mastication, sleeping position, oral habits, breathing, nutrition, and crossbite, are suspected to affect the development of craniofacial or occlusal asymmetry (2–8).

However, perfect facial symmetry is not common, and all faces have some degree of asymmetry. Normal asymmetry in a population can be directional or fluctuating (5). Fluctuating asymmetry is a random deviation from perfect symmetry, whereas directional asymmetry in the human body manifests, for example, in the laterality of organs and handedness. Normal directional asymmetry is suspected to arise from left–right differences in brain and skull base structures (5,9,10). Attempts have been made to identify a direction and a dominant side of asymmetry in the facial area (10–13). The results have varied, possibly due to different study methods and imaging projections, difficulties in distinguishing directional asymmetry from fluctuating asymmetry and the fact that multiple etiologic factors may affect the development of facial asymmetry.

Obviously, the majority of facial asymmetries require no treatment. Nevertheless, prominent facial asymmetry might be a significant issue for an orthodontic patient. When treatment is needed, asymmetry might be challenging to correct and require complex orthodontic and surgical treatment. To date, no thresholds have been set to distinguish between normal and increased asymmetry. Three-dimensional (3D) soft tissue imaging enables reproducible measurement of facial asymmetry as well as following the development of facial asymmetries or growth disturbances. Moreover, 3D imaging might be sophisticated enough to analyse the subtle effects of various minor factors that might affect the development of facial asymmetry.

Longitudinal studies analysing the development of facial asymmetry in a normal cohort are rare. Before modern 3D imaging methods, most longitudinal studies used either photography or X-ray-based two-dimensional (2D) imaging methods (14,15). 3D imaging methods enable studying complex craniofacial structures in three dimensions. Currently, cone-beam computer tomography is the predominant method for studying facial structures (16). However, as an X-ray-based method, it includes the potential risk of ionising radiation, and thus its use is based on a risk-benefit assessment (17). Hence, the use of the method is limited to special medical care, and it is not suitable for longitudinal studies with normal populations, particularly children. During the past few decades, methods have been developed for imaging and analysing photography-based 3D facial images. Some longitudinal 3D-based studies on facial growth and the development of facial asymmetry in normal populations have been published (18–22).

This study aimed to investigate the development of facial asymmetry in individuals up to 6 years of age using the 3D soft tissue imaging method in a normal population. As a second aim, the study sought to identify potential predisposing factors to facial asymmetry.

## Materials and methods

### Participants

This prospective, population-based cohort study was carried out in the Research Unit of Oral Health Sciences, the University of Oulu and the Clinic for Children and Adolescents, Oulu University Hospital. Approval was obtained from the ethics committee of the Northern Ostrobothnia Hospital District (Oulu University Hospital; EETTMK 27/2011). Written informed consent was obtained from all of the parents of the participants.

Participants were recruited at Oulu University Hospital. The recruitment dates, spread throughout the year, were pre-selected for the period between February 2012 and December 2013. Participants born on those pre-selected dates were included in the eligibility assessment. The inclusion criteria were as follows: born full-term (after 37 weeks of gestation), healthy enough to maintain without intensive care, and resided within a 30-minute driving distance from Oulu University Hospital. Exclusion criteria were a diagnosis of cheilopalatoshisis, craniosynostosis, or other dysmorphic features. A total of 102 newborns were enrolled. All participants were part of a randomized controlled trial (RCT) (23). The study was registered in the National Clinical Trials register (NCT02283229). In the trial, the infants’ parents in the intervention group received special counselling to prevent the development of deformational plagiocephaly (DP). The RCT setting was terminated at the age of 3 months, and all participants received advice if needed. All the children who participated in the previous trial or the later follow-up studies (20,24,25) were re-invited to this study.

### Data acquisition

All participants were examined at birth and underwent examination and 3D stereophotogrammetric imaging of the head and face at the ages of approximately 3 months, 6 months, 12 months, 3 years, and 6 years. However, in the present study, only data at the ages of 12 months (T1), 3 years (T2), and 6 years (T3) were used in the facial symmetry analysis. During the imaging procedure, a tight nylon sock cap was fitted on each subject’s head, with hair from the forehead placed inside the cap. Subjects were set on an adjustable chair at a standard distance from the cameras. If needed, a parent assisted each young child to stabilize and centre the head optimally during imaging. Facial images were obtained in a natural head position with the jaw relaxed (26). Images were evaluated immediately after imaging, and a new image was captured if necessary.

Background data regarding pregnancy and delivery were collected from maternal and infant medical records. In addition, the infants’ parents filled out a questionnaire regarding care habits at each visit during the first 3 years of follow-up.

Dental occlusion was examined and registered at the age of 3 and 6 years. For sagittal occlusion, the anterior crossbite was registered, and the sagittal molar relationship on both sides was defined according to the Angle classification. Sagittal occlusion of the molars was classified as neutral, mesial, or distal based on half-cusp accuracy. For transversal occlusion, crossbite, scissor bite, and deviation (mm) of the dental midline from the facial midline were registered. Crossbite was registered if at least one maxillary posterior tooth had a buccal cusp occluding lingually to the buccal cusp of a mandibular tooth (27,28). Occlusion was determined to be asymmetric if a subject had a deviation of dental midlines 2 mm or greater, an asymmetric angle classification between the right and left sides, or a unilateral crossbite.

### 3D analysis methods

**Figure 1:**
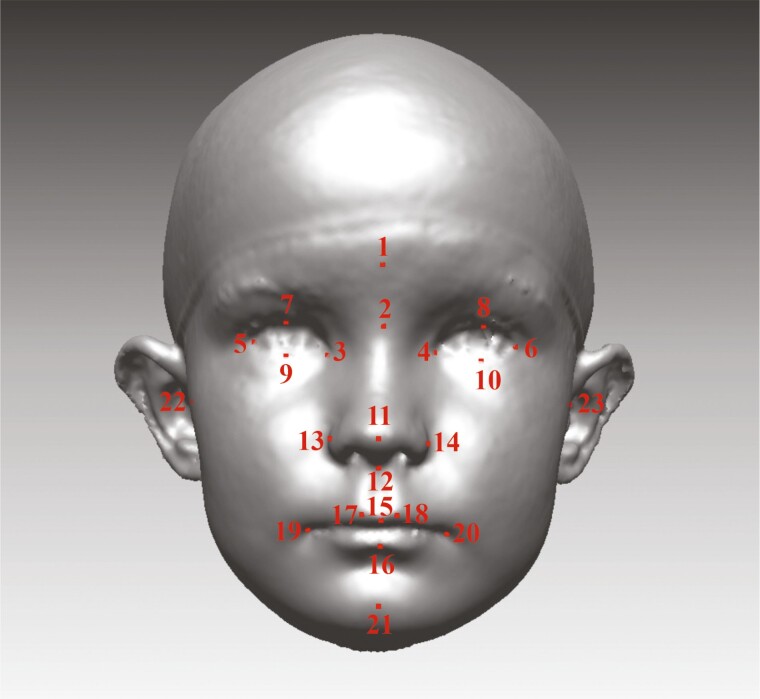
The Farcas soft tissue landmarks: 1 Glabella (g); 2 Nasion (n); 3, 4 Endocanthion (en); 5, 6 Exocanthion (ex); 7, 8 Pulpabrale superius (ps); 9, 10 Pulpabrale inferius (pi); 11 Pronasale (prn); 12 Subnasale (sn); 13, 14 Alare (al); 15 Labiale superius (ls); 16 Labiale inferius (li); 17, 18 Christa philtra (cph); 19, 20 Cheilion (ch); 21 Pogonion (pg); and 22, 23 Tragion (Tr).

Facial images at the ages of 12 months (T1), 3 years (T2), and 6 years (T3) were processed and analysed with the Rapidform 2006 (Geomagic, Rock Hill, South Carolina, USA) 3D software system using custom macros written with Visual Basic for Applications. More complex mathematical analyses were performed with MATLAB R2014b (MathWorks, Natick, Mississippi, USA). All distinct parts were manually removed from each image, maintaining the widest possible area of the face. For each image, a total of 23 soft tissue landmarks (29,30) were manually identified by one author (Figure 1). The facial position of each image was standardised, as proposed by Zhurov *et al*. (31). Next, all the facial images were scaled to the same size based on the average centroid size (Frobenius norm of landmark matrix, in which landmark coordinates are in rows). Scaling the images to the same size eliminates errors caused by the growth and size differences.

**Figure 2:**
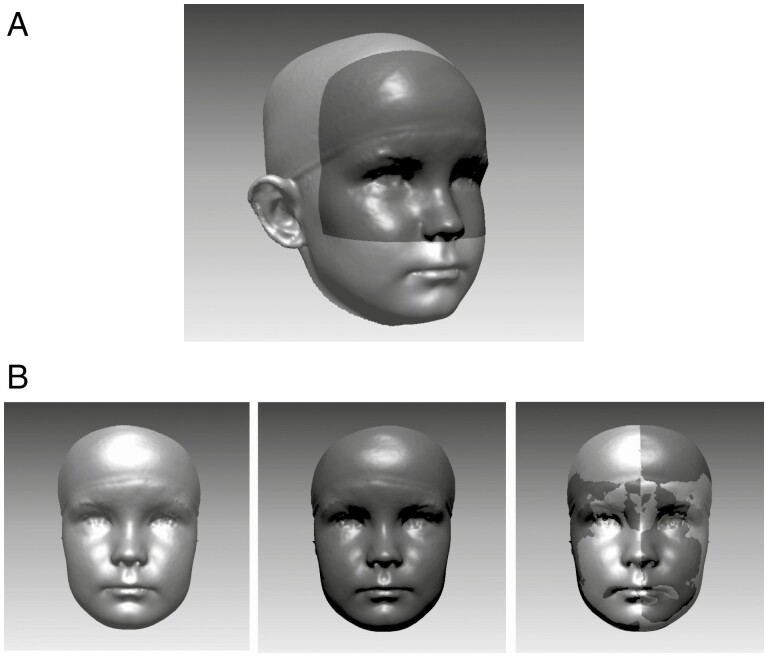
The superimposition procedure. a) Dark grey indicates the area used in the superimposition of the images; an area limited vertically to the subnasion and sagittally to the midpoint between the exocanthion and tragion was used. b) An original image, a mirrored image and the original image superimposed with the mirrored image.

To determine the sagittal midline, the facial surface was mirrored across the sagittal plane. Next, a special area limited vertically to the subnasion and sagittally to the midpoint between the exocanthion and tragion was determined (Figure 2). Subsequently, the two surfaces were superimposed using the iterative closest point algorithm on the special area described above. The facial midline was thus formed on the line of symmetry of the superimposed image. The delimited area was used in the superimposition due to the concern that the lower jaw and temple areas might cause inaccuracy on the facial midline.

**Figure 3:**
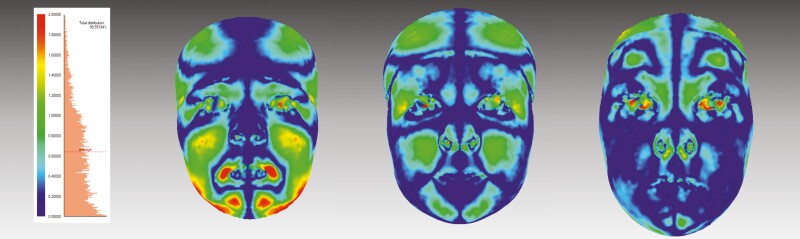
A demonstration of landmark-independent method: facial asymmetry on colour deviation maps of one participant at the ages of 12 months, 3 years, and 6 years. Colours indicate the magnitude of the difference between the original and mirrored facial shell.

Each facial image was divided into the four following areas: the upper face (above the endocanthion line), the upper mid-face (between the endocanthion line and the subnasal), the lower mid-face (between the subnasal and the cheilion line), and the lower face (under the cheilion line). The average distance (mm) and the percentage of symmetry (%) between the original and mirrored face were calculated for each facial area separately (Figure 3). The symmetry percentage was calculated as the face area where the distance between the original face and the mirrored surface did not exceed 0.5 mm (32,33).

The distance of facial landmarks to the facial midline was analysed. For paired landmarks, the midpoint of the two landmarks was analysed. Both true values and corresponding absolute values were measured. Landmarks were assigned positive true values if they were located on the person’s left side of the facial midline and negative values if they were located on the right side.

To determine cranial asymmetry, the Anterior Cranial Asymmetry Index (ACAI) and the Oblique Cranial Length Ratio (OCLR) were measured from the 3D images. A cut-off value of 104% for OCLR was used for the diagnosis of DP (25,34).

### Statistical analysis

IBM SPSS Statistics for Windows, Version 25.0 (IBM SPSS Statistics, Armonk, New York, USA, IBM Corp.) was used for statistical analysis.

A repeated measures ANOVA was used to analyse the development of facial symmetry parameters in the study cohort from T1 to T3. Bonferroni corrections were used in the *post hoc* analysis. Both surface-based and landmark-based methods were used. One sample *t*-test was used to test the actual deviation of landmarks from the facial midline in the right–left direction. A paired sample *t*-test was used to compare the average values of all lower facial landmarks to upper facial landmarks. A descriptive analysis was performed for dental occlusion as well as occlusal asymmetries at the ages of 3 and 6 years. An independent sample *T*-test was used to test facial landmarks between children with and without asymmetric right-deviating occlusions at T3. To investigate potential explanatory variables for facial asymmetry at T3, independent sample *t*-tests and simple linear regressions were performed. Finally, multiple linear regression models were run separately for each facial part (upper face, upper mid-face, lower mid-face, and lower face). A stepwise strategy was used to select variables for the final models (inclusion criteria *P* <0.05, exclusion criteria *P* >0.10). For each facial part, variables with *P* <0.2 in the independent sample *t*-test or simple linear regressions were proposed for inclusion in the multiple linear regression models.

## Results

### The course of facial symmetry

**Figure 4:**
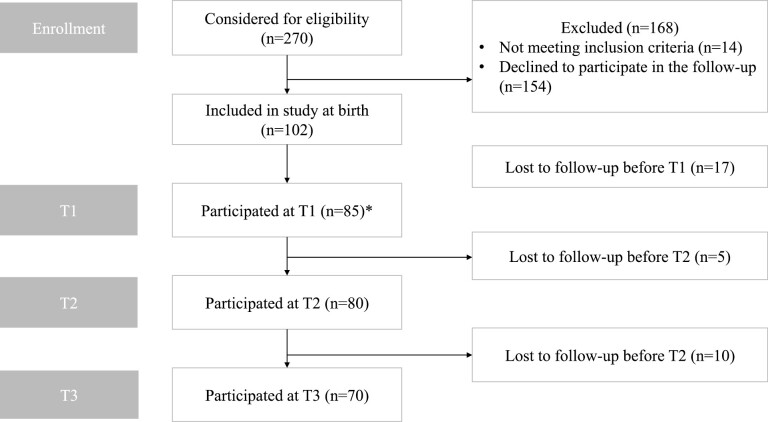
Flowchart of the children in the study at the enrolment at birth and at the age of 12 months (T1), 3 years (T2), and 6 years (T3). * = At T1, five images were excluded because a child was moving or crying.

**Figure 5:**
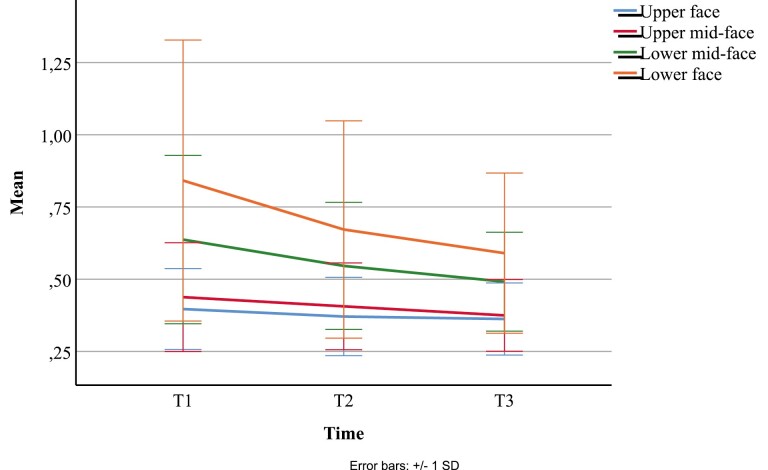
The course of facial asymmetry: Mean AvD (mm) for upper face (blue line), upper mid-face (red line), lower mid-face (green line), and lower face (orange line) at T1 (12 months old), T2 (3 years old), and T3 (6 years old). Error bars represent standard deviations of each value.

The study flow is presented in Figure 4. A total of 70 (68.6%) subjects participated in T3. There were 31 (44.3%) female and 39 (55.7%) male participants. The mean (SD) ages at T1, T2, and T3 were 1.01 (0.04), 3.02 (0.14), and 6.01 (0.16) years, respectively. Table 1 presents the course of facial symmetry in this study cohort from T1 to T3. As measured by surface-based variables, facial symmetry improved between T1 and T3 in all facial areas. Average distance (AvD) decreased in the whole face area from 0.52 to 0.43 mm (*P* < 0.001), in the upper face area from 0.40 to 0.36 mm (*P* = 0.037), in the upper ­mid-face area from 0.44 to 0.37 mm (*P* = 0.002), in the lower mid-face area from 0.64 to 0.49 mm (*P* = 0.003), and in the lower face area from 0.84 to 0.59 (*P* < 0.001). Landmark-based linear values (distance of landmarks from facial midline) did not differ significantly between T1 and T3, except for the Cheilion. No statistically significant differences were found between genders in any of the facial symmetry parameters reported in Table 1. A graphical presentation of the development of the average distance from 1 year (T1) to 6 years (T3) is shown in Figure 5. The mean AvD tends to decrease in all facial parts from T1 to T3. Figure 5 also shows a wide standard deviation, representing large individual variations in these parameters. The standard deviation was the largest on the lower face. Further, the variation was greater at an earlier age and decreased with time.

**Table 1.** The course of facial symmetry measured by surface-based variables [average distance (mm) and symmetry percentage (%)] and landmark-based linear variables [landmark distance from the facial midline (mm)] at T1, T2, and T3. SD = standard deviation; IQR = interquartile range; CI = confidence interval; * = statistical significance at the *P* < 0.05 level (*P*^a^-value = *A repeated measures ANOVA* was used, *P*^b^-value = Bonferroni corrections was used).

												*Post hoc* analysis											
		T1			T2			T3				T1–T2				T1–T3				T2–T3			
														95% CI				95% CI				95% CI	
Surface-based variables		Mean (SD)	Median	IQR	Mean (SD)	Median	IQR	Mean (SD)	Median	IQR	*P* ^a^-value	*P* ^b^-value		Lower	Upper	*P* ^b^-value		Lower	Upper	*P* ^b^-value		Lower	Upper
Average distance (mm)																							
Whole face		0.52 (0.17)	0.48	0.39–0.62	0.46 (0.14)	0.43	0.37–0.54	0.43 (0.10)	0.43	0.35–0.49	0.000	0.040		0.00	0.11	0.000	*	0.04	0.14	0.129		–0.01	0.07
Upper face		0.40 (0.14)	0.34	0.30–0.45	0.37 (0.14)	0.33	0.29–0.48	0.36 (0.12)	0.34	0.27–0.41	0.022	0.183	*	–0.01	0.06	0.037	*	0.00	0.07	1.000		–0.02	0.04
Upper mid-face		0.44 (0.19)	0.40	0.31–0.51	0.41 (0.15)	0.37	0.29–0.48	0.37 (0.12)	0.35	0.27–0.45	0.001	0.216		–0.01	0.07	0.002	*	0.02	0.11	0.108		–0.00	0.07
Lower mid-face		0.64 (0.29)	0.58	0.46–0.72	0.55 (0.22)	0.50	0.38–0.65	0.49 (0.17)	0.47	0.36–0.57	0.002	0.105		–0.01	0.20	0.003	*	0.04	0.25	0.184		–0.02	0.13
Lower face		0.84 (0.49)	0.65	0.49–1.06	0.67 (0.38)	0.53	0.37–0.85	0.59 (0.28)	0.51	0.39–0.76	0.001	0.069		–0.01	0.35	0.000	*	0.10	0.40	0.305		–0.04	0.20
Symmetry percentage (%)																							
Whole face		65.3 (10.15)	65.80	61.09–71.97	67.79 (10.74)	69.05	60.62–75.48	69.29 (9.95)	69.06	63.25–77.12	0.015	0.283		–6.08	01-Nov	0.024	*	–7.58	–0.40	0.597		–4.34	Jan-34
Upper face		72.78 (13.2)	75.88	65.68–82.96	74.57 (14.71)	78.53	63.02–83.54	74.9 (13.92)	77.58	68.91–85.54	0.247												
Upper mid-face		72.24 (12.39)	73.43	61.69–81.76	72.86 (13.97)	73.94	64.65–85.28	75.68 (13.91)	78.31	65.93–87.74	0.057												
Lower mid-face		55.52 (17.9)	55.79	43.96–68.94	59.32 (16.39)	58.81	48.60–72.97	62.42 (16.34)	61.69	50.63–75.15	0.043	0.552		–10.77	Mar-16	0.063		–14.10	0.28	0.507		–8.59	Feb-38
Lower face		43.93 (19.89)	43.92	27.36–60.82	52.08 (23.22)	54.00	28.52–71.92	55.11 (21.08)	56.09	37.40–69.69	0.005	0.071		–16.79	0.49	0.003	*	–19.14	–3.22	1.000		–11.93	May-87
Landmark-based linear variables	Distance from the facial midline (mm)																						
Upper face	G	0.54 (0.41)	0.42	0.18–0.81	0.68 (0.45)	0.69	0.29–0.97	0.68 (0.49)	0.60	0.28–0.94	0.090												
	N	0.38 (0.29)	0.37	0.10–0.61	0.44 (0.33)	0.40	0.14–0.66	0.39 (0.31)	0.33	0.16–0.53	0.443												
Eye area	Mid-Ps	0.60 (0.45)	0.53	0.20–0.84	0.54 (0.41)	0.50	0.18–0.83	0.50 (0.38)	0.41	0.23–0.68	0.361												
	Mid-En	0.31 (0.22)	0.28	0.14–0.43	0.33 (0.25)	0.27	0.11–0.49	0.39 (0.27)	0.35	0.18–0.57	0.139												
	Mid-Ex	0.46 (0.34)	0.48	0.12–0.66	0.49 (0.32)	0.42	0.28–0.71	0.52 (0.44)	0.44	0.14–0.69	0.574												
	Mid-Pi	0.68 (0.48)	0.51	0.33–1.04	0.66 (0.43)	0.63	0.26–1.00	0.53 (0.4)	0.43	0.20–0.78	0.102												
Nose area	Prn	0.29 (0.26)	0.26	0.08–0.39	0.34 (0.28)	0.27	0.13–0.52	0.36 (0.28)	0.32	0.13–0.50	0.242												
	Mid-Al	0.27 (0.21)	0.21	0.10–0.39	0.22 (0.18)	0.18	0.09–0.34	0.27 (0.21)	0.25	0.10–0.35	0.222												
	Sn	0.33 (0.28)	0.24	0.12–0.47	0.31 (0.27)	0.25	0.10–0.48	0.32 (0.29)	0.25	0.08–0.47	0.900												
Lip area	Mid-Cph	0.61 (0.5)	0.50	0.19–0.89	0.64 (0.52)	0.43	0.25–1.05	0.57 (0.44)	0.46	0.19–0.86	0.636												
	Ls	0.54 (0.49)	0.38	0.17–0.74	0.57 (0.46)	0.48	0.19–0.76	0.47 (0.38)	0.37	0.11–0.74	0.376												
	Mid-Ch	0.95 (0.73)	0.76	0.37–1.46	0.65 (0.56)	0.48	0.18–0.95	0.60 (0.44)	0.54	0.26–0.85	0.001	0.014		0.048457	0.556256	0.004		0.092545	0.599868	1.000		–0.173537	0.261237
	Li	0.82 (0.81)	0.55	0.25–1.16	0.65 (0.56)	0.53	0.23–0.87	0.57 (0.45)	0.43	0.19–0.87	0.056												
Chin area	Pg	1.23 (1.27)	0.80	0.33–1.75	0.99 (0.88)	0.71	0.32–1.21	0.89 (0.70)	0.76	0.31–1.40	0.069												
Tragus area	Mid-Tr	0.67 (0.51)	0.56	0.31–0.99	0.59 (0.43)	0.46	0.23–0.89	0.57 (0.45)	0.50	0.20–0.84	0.144												

Table 2 presents the relationship of the facial landmarks to the facial midline. The presented true values indicate the actual deviation of each landmark from the facial midline in the right–left direction. Lower facial landmarks were located significantly more on the right side, while upper facial landmarks were located more on the left side in relation to the facial midline: The mean values for landmarks in the upper face (Glabella and Nasion), as well as mid-landmarks in eye areas (Mid-Pulpabrale superius, Mid-Endocanthion, Mid-Exocanthion, and Mid-Pulpabrale inferius) differed significantly from the facial midline, with positive values at T1, T2, and T3 (*P* < 0.05). On the contrary, the mean values for landmarks located in the middle and lower facial area, Mid-Alare, Mid-Christa Philtra, and Mid-Cheilion, differed significantly from the facial midline with negative values at T1, T2, and T3 (*P* < 0.05). In other facial landmarks, the individual variation was large and therefore did not achieve statistical significance.

**Table 2.** The relationship of landmarks from the facial midline at T1 (*N* = 65), T2 (*N* = 70), and T3 (*N* = 70) presented as true values (mm). Landmarks receiving negative mean values are located on the person’s right side in relation to the facial midline, and positive values are located on the left side. SD = standard deviation; CI = confidence interval; * = statistical significance at the *P* < 0.05 level (*P*-value = one-sample *t*-test was used).

		T1 (*N* = 65)					T2 (*N* = 70)					T3 (*N* = 70)				
					95% CI					95% CI					95% CI	
		Mean (SD)	*P*-value		Lower	Upper	Mean (SD)	*P*-value		Lower	Upper	Mean (SD)	*P*-value		Lower	Upper
Upper face	G	0.41 (0.54)	<0.001	*	0.28	0.55	0.60 (0.55)	<0.001	*	0.47	0.73	0.52 (0.65)	<0.001	*	0.36	0.67
	N	0.24 (0.41)	<0.001	*	0.14	0.35	0.37 (0.41)	<0.001	*	0.27	0.47	0.28 (0.41)	<0.001	*	0.18	0.38
Eye area	Mid-Ps	0.30 (0.69)	0.001	*	0.13	0.47	0.18 (0.67)	0.025	*	0.02	0.34	0.26 (0.57)	<0.001	*	0.13	0.40
	Mid-En	0.10 (0.37)	0.031	*	0.01	0.19	0.25 (0.32)	<0.001	*	0.18	0.33	0.28(0.36)	<0.001	*	0.20	0.37
	Mid-Ex	0.22 (0.53)	0.001	*	0.09	0.35	0.36 (0.46)	<0.001	*	0.25	0.47	0.38 (0.54)	<0.001	*	0.25	0.51
	Mid-Pi	0.50 (0.68)	<0.001	*	0.33	0.67	0.51 (0.61)	<0.001	*	0.37	0.66	0.38 (0.53)	<0.001	*	0.26	0.51
Nose area	Prn	–0.04 (0.39)	0.469		–0.13	0.06	0.14 (0.42)	0.007	*	0.04	0.24	0.04 (0.45)	0.498		–0.07	0.14
	Mid-Al	–0.15 (0.31)	<0.001	*	–0.22	–0.07	-0.08(0.27)	0.016	*	–0.14	–0.01	-0.09 (0.33)	0.025	*	–0.17	–0.01
	Sn	–0.02 (0.43)	0.725		–0.13	0.09	–0.06 (0.4)	0.241		–0.15	0.04	0.030 (0.43)	0.564		–0.07	0.13
Lip area	Mid-Cph	–0.31 (0.73)	0.001	*	–0.49	–0.13	–0.32 (0.76)	0.001	*	–0.50	–0.14	-0.30 (0.64)	<0.001	*	–0.45	–0.14
	Ls	0.00 (0.74)	0.978		–0.19	0.18	0.05 (0.72)	0.555		–0.12	0.22	0.13 (0.59)	0.081		–0.02	0.27
	Mid-Ch	–0.49 (1.10)	0.001	*	–0.76	–0.22	–0.37 (0.8)	<0.001	*	–0.56	–0.18	–0.17 (0.72)	0.047	*	–0.34	0.00
	Li	–0.01 (1.15)	0.938		–0.30	0.27	0.09 (0.84)	0.357		–0.11	0.29	0.09 (0.72)	0.308		–0.08	0.26
Chin area	Pg	–0.35 (1.74)	0.112		–0.78	0.08	–0.14 (1.3)	0.38		–0.45	0.17	–0.24 (1.1)	0.077		–0.50	0.03
Tragus area	Mid-Tr	–0.13 (0.84)	0.207		–0.34	0.08	–0.13 (0.78)	0.164		–0.32	0.06	–0.15 (0.73)	0.098		–0.32	0.03

The average value of all lower facial landmarks (Labiale superius, Labiale inferius, Christa philtra, Cheilion, and Pogonion) was significantly more negative than the average value of all upper and mid-facial landmarks (Glabella, Nasion, Endocanthion, Exocanthion, Pulpabrale superius, Pulpabrale inferius, Pronasale, and Subnasale, Alare) at T1, T2, and T3. At T1, the mean (SD) distance from the facial midline for lower facial landmarks was –0.23 (0.96) mm, and that for the upper and mid-facial landmarks was 0.18 (0.26) mm (*P* = 0.001). At T2, the mean (SD) distance from the facial midline for lower facial landmarks was –0.11 (0.73) mm, and that for the upper and mid-facial landmarks was 0.25 (0.25) mm (*P* < 0.001). At T3, the mean (SD) distance from the facial midline for lower facial landmarks was –0.13 (0.63) mm, and that for the upper and mid-facial landmarks was 0.24 (0.21) mm (*P* < 0.001).

The occlusal deviations were found to be consistent with soft tissue deviations. As shown in Table 3, the majority of asymmetries in the occlusions were found to be towards the right side, indicating a tendency of slight lower jaw deviation to the right. Unilateral crossbites were found on the right side at the ages of 3 and 6 years. Additionally, lower midline deviations were more often found on the right side; at the age of 3 years, all five midline deviations were towards the right side, and at the age of 6 years, 10 out of 12 midline deviations were towards the right side. At the age of 3 years, four (6%) children had an asymmetric molar relationship, three of which were towards the right side (the relationship was more distal on the right side). At the age of 6 years, eight (12%) children had an asymmetric molar relationship, seven of which were towards the right side. All occlusal characteristics are presented in Table 3. At T3, a total of 17 subjects had asymmetric occlusion deviating to the right, and five subjects had asymmetric occlusion deviating to the left. No statistically significant differences were found in facial landmarks between children with and without asymmetric right-deviating occlusions at the age of 6 years.

**Table 3.** Descriptive analysis of occlusal characteristics at T2 and T3 *N* (%).

Occlusal characteristics		T2	T3
Molar relationship			
	Neutral	46 (71%)	44 (67%)
	Distal	12 (18%)	7 (10.5%)
	Mesial	3 (5%)	7 (10.5%)
	Asymmetric molar relationship	4 (6%)	8 (12%)
Asymmetric occlusion	Asymmetric molar relationship		
	Deviating to right	3	7
	Deviating to left	1	1
	Asymmetric canine relationship		
	Deviating to right		12
	Deviating to left		0
	Deviation of lower midline (over 2 mm)		
	Deviating to right	5	10
	Deviating to left	0	2
	Deviation of upper midline (over 2 mm)		
	Right		4
	Left		3
	Crossbite		
	Right	1	3
	Left	0	0
	Asymmetry in occlusion	9	22
	Deviating to right	8	17
	Deviating to left	1	5

### Predisposing factors for facial asymmetry

Independent sample *t*-tests and simple linear regression models were performed to test whether different variables predicted facial asymmetry (measured as AvD) at T3. All tested variables are presented in Table 4. The results of the simple linear regression are reported in Supplementary Table 1.

**Table 4.** Characteristics of putative variables proposed to be included in multiple linear regression models *N* (%) or mean (SD).

	*N* (%) or mean (SD)
Male sex	39 (57)
BMI 6 years	15.8 (1.2)
Preventive parental counselling	35 (50)
DP history	22 (31)
Positional preference at 3 or 6 months	10 (14)
Sleeping position	
Primary supine 3 months (*N* = 58)	39 (67)
Primary supine 6 months (*N* = 69)	45 (65)
Primary supine 12 months (*N* = 62)	12 (63)
Acute otitis media (one or more) (*N* = 61)	41 (67)
Occlusion	
Asymmetric occlusion 3 years	9 (13)
Asymmetric occlusion 6 years	22 (31)
Breastfeeding duration (*N* = 64)	
Not at all or under 3 months	4 (6)
3–6 months	10 (16)
6–12months	25 (39)
12 months or over	25 (39)
Pacifier use duration (*N* = 64)	
Not at all or under 3 months	19 (27)
3–6 months	2 (3)
6–12months	15 (21)
12 months or over	34 (49)
OCLR	
3 months (*N* = 70)	102.7 (2.4)
6 months (*N* = 69)	102.4 (2.3)
12 months (*N* = 69)	102.2 (1.9)
3 years (*N* = 68)	102.1 (1.6)
6 years (*N* = 70)	101.9 (1.5)
ACAI	
3 months (*N* = 70)	2.5 (1.8)
6 months (*N* = 69)	2.1 (1.7)
12 months (*N* = 69)	2.1 (1.6)
3 years (*N* = 68)	1.9 (1.4)
6 years (*N* = 70)	1.8 (1.4)
Head rotation and flexion	
Imbalance in head rotation, 0 months, (*N* = 70)	4.1 (3)
Imbalance in lateral flexion, 0 months, (*N* = 70)	3.5 (2.6)
Imbalance in head rotation, 3 months, (*N* = 70)	4.2 (4.1)
Imbalance in lateral flexion, 3 months, (*N* = 70)	5.2 (4.8)
Imbalance in head rotation, 6 months, (*N* = 70)	4.3 (3.9)
Imbalance in lateral flexion, 6 months, (*N* = 70)	3.3 (3.4)

#### Earlier facial asymmetry

Facial asymmetry at T2 was one of the strongest predictors of facial asymmetry at T3. It was found that AvD at T2 significantly predicted AvD at T3 for the upper face (β = 0.69, *P* < 0.001, 95% CI = 0.55–0.84), upper mid-face (β = 0.54, *P* < 0.001, 95% CI = 0.40–0.68), lower mid-face (β = 0.23, *P* = 0.010, 95% CI = 0.06–0.40), and lower face (β = 0.23, *P* = 0.010, 95% CI = 0.06–0.40).

#### Cranial asymmetry

Previous cranial asymmetry predicted facial asymmetry on all facial parts except for the lower face. Children with a history of DP had higher scores for AvD at T3 for the upper face (0.41 versus 0.33, *P* = 0.013), upper mid-face (0.43 versus 0.35, *P* = 0.015), and lower mid-face (0.55 versus 0.46, *P* = 0.044).

Similarly, upper face asymmetry at T3 was associated with OCLR at 3 months (*P* = 0.007), 6 months (*P* = 0.010), and 12 months (*P* = 0.015). Upper mid-face asymmetry at T3 was associated with OCLR at 3 months (*P* = 0.002), 6 months (*P* = 0.004), 12 months (*P* = 0.001), 3 years (*P* = 0.015), and 6 years (*P* = 0.034), while lower mid-face asymmetry at T3 was associated with OCLR at 3 months of age (*P* = 0.045).

Also, ACAI significantly predicted upper facial asymmetry. AvD in the upper facial area at T3 was associated with ACAI at 3 months (*P* < 0.001), 6 months (*P* = 0.007), 12 months (*P* = 0.031), and 3 years (*P* = 0.034) of age. AvD in the upper mid-face area at T3 was associated with ACAI at 3 years (*P* = 0.04) and 6 years (*P* = 0.005) of age.

#### Lateral flexion or rotation

A few but opposing associations were found between AvD at T3 and lateral flexion or rotation imbalance during infancy. An imbalance in head rotation or flexion was tested at birth, at 3 months and at 6 months of age. An imbalance in lateral flexion at birth predicted upper facial symmetry at T2 (β = –0.01, *P* = 0.034, 95% CI = –0.02–0.00), an imbalance in lateral flexion at the age of 3 months predicted lower facial symmetry at T2 (β = –0.02, *P* = 0.012, 95% CI = –0.03–0.00) and an imbalance in head rotation at the age of 6 months predicted upper mid-face asymmetry at T2 (β = 0.01, *P* = 0.023, 95% CI = 0.00–0.02).

#### Environmental factors

Of the environmental factors, sleeping position and breastfeeding duration were found to have connections with facial symmetry at T3. Those who slept primarily in the supine position at the age of 6 months had lower scores for AvD at T3 for the lower mid-face (0.44 versus 0.57, *P* = 0.001) and lower face (0.50 versus 0.74, *P* < 0.001). Further, breastfeeding duration predicted facial symmetry in the upper mid-face (β = –0.04, *P* = 0.027, 95% CI = –0.07–0.00) and lower mid-face (β = –0.05, *P* = 0.043, 95% CI = –0.10–0.00). The duration of pacifier use was not associated with facial asymmetry measured by AvD.

Sex, body mass index at the age of 6 years, a lack of parental counselling at birth, acute otitis media in infancy, and positional preference at the ages of 3 or 6 months were not associated with AvD at T3 in any facial areas.

No connection was found between asymmetric occlusion at the ages of 3 or 6 months and the AvD at T3 in any facial area.

### Multiple linear regression model

Multiple linear regression models for AvD at T3 for the upper face, upper mid-face, lower mid-face, and lower face are shown in Table 5. For the upper face, AvD at T2 significantly predicted the value at T3 (*P* < 0.001). For the upper mid-face, AvD at T3 was significantly predicted by AvD at T2 (*P* < 0.001), ACAI at 6 months (*P* = 0.004), and an imbalance in head rotation at the age of 6 months (*P* = 0.010), whereas supine sleeping at 12 months was associated with a reduction in upper mid-face AvD at T3 (*P* = 0.015). For the lower mid-face, a history of DP predicted AvD (*P* = 0.021) and supine sleeping at 6 months reduced (*P* = 0.002) AvD at T3. Finally, for the lower face, a significantly predictive variable was AvD at T2 (*P* = 0.027), while supine sleeping at 6 months was associated with a reduction in AvD at T3 (*P* = 0.001).

**Table 5.** Results of the multiple linear regression for each facial part (the upper face, upper mid-face, lower mid-face, and lower face) estimating different factors affecting facial asymmetry, measured as AvD at T3. CI = confidence interval, *adjusted for other variables included in the model.

	Average distance upper face		Average distance upper mid-face		Average distance lower mid-face		Average distance lower face	
Independent variable	Coefficient (95% CI)*	*P*-value	Coefficient (95% CI)*	*P*-value	Coefficient (95% CI)*	*P*-value	Coefficient (95% CI)*	*P*-value
Value of dependent variable at T2	0.63 (0.45–0.81)	<0.001	0.40 (0.26–0.55)	<0.001			0.18 (0.02–0.34)	0.027
History of DP					0.11 (0.02–0.20)	0.021		
ACAI 6 months			0.02 (0.01–0.04)	0.004				
Supine sleeping 6 months					–0.14(–0.23––0.06)	0.002	–0.21 (–0.34––0.09)	0.001
Supine sleeping 12 months			–0.07 (–0.12––0.01)	0.015				
Imbalance in head rotation, 6 months			0.01 (0.00–0.01)	0.010				

## Discussion

This study investigated the prevalence and development of facial asymmetry in young children up to 6 years of age. Facial asymmetry was already present in early childhood in a normal population. However, it reduced significantly between 1 and 6 years. The present study complements earlier studies reporting the development of facial asymmetry in children in other age groups. Primozic *et al*. (18). studied children 5 to 10 years of age, and Djordjevic e*t al*. (19). examined children 11–16 years of age. Both reported that facial asymmetry did not tend to increase or decrease. The course of craniofacial asymmetry up to the age of 3 years in this same study population was reported earlier (20). During that period, symmetry improved both in the cranial and the upper facial areas (20). Although facial symmetry tends to either improve or remain the same during growth at a population level, there might be significant individual variation and fluctuation during growth. Liukkonen *et al*. longitudinally studied mandibular asymmetry in 2D projections and concluded that mandibular growth might fluctuate throughout childhood (35). Similarly, Melnik *et al*. (14). found that mandibular asymmetry could diminish or appear during growth. In the present study, a large individual variation was found in facial symmetry parameters. However, the variation increased the younger the children were. Moreover, the variation was larger on the lower parts of the face, where facial expressions have a higher effect on the accuracy of the measurements. Apparently, lower jaw stability increases as the child ages and more teeth erupt to occlusion.

Whereas the surface-based method allows accurate analysis of the development of asymmetry throughout the whole facial area, the landmark-based analysis provides additional information on asymmetry in the most eye-catching parts of the face. Moreover, the landmark-based method, combined with surface-based midline determination, enables an accurate study of the direction of facial asymmetry in three dimensions. Similar to the surface-based method, the landmark-based analysis also showed significant asymmetry at an early age. Interestingly, the landmark-based analysis showed that upper facial landmarks were more commonly located on the left of the facial midline, while lower facial landmarks were more commonly located on the right of it. The results indicate slight directional asymmetry in this population at an early age. Moreover, most of the asymmetric occlusal deviations of the lower jaw were towards the right side, although no statistically significant association was found between the direction of occlusal and facial asymmetry. However, no differences were found in landmark deviations between children with and without asymmetric right-deviating occlusions. Before 3D imaging, an attempt has been made to find the direction of facial asymmetry. Photographs and X-rays taken from different projections have been used as research methods, with the majority of the studies involving adult patients. However, the results have been contradictory, probably due to the different methods and difficulty in determining the facial midline. Based on posteroanterior radiographs, some studies have reported that the left side of the face is wider (11,12), while others have indicated that the right side is larger (13). Photography-based studies of facial soft tissue asymmetry have usually reported that the left side of the face is wider (36–38).

Multiple etiological factors affect the development of facial asymmetry. Therefore, the present study sought to analyse potential predisposing factors for facial asymmetry in a normal population. However, because of the cohort-based study design, the incidence of all factors was minor. Thus, this investigation rather examines how the impact of many minor factors could be detected with 3D facial imaging methods.

Cross-sectional 3D facial studies have reported a connection between DP and facial asymmetry (39–42). This connection was also seen in the present cohort follow-up during infancy and toddler ages. However, as the prevalence of DP and cranial asymmetry diminished over the first 3 years of life, the effect of DP on facial asymmetry also seemed to reduce during growth in early childhood (20). This was the case in our study population, which was cohort-based, and most of the DP cases were mild. In the present study, both simple and multiple linear regression models showed that cranial asymmetry and DP during the first months of life predisposed children to facial asymmetry at the age of 6 years. Nevertheless, neither known risk factors for DP, such as positional preference (24), nor protective factors for DP, such as preventive parental counselling (23), seemed to be associated with facial asymmetry later in life.

The fact that the incidence of DP has increased after the recommendation that infants sleep in a supine position to prevent sudden infant death syndrome (43) has led to more extensive investigations of the effect of a one-sided sleeping position on the postnatal development of cranial asymmetry. The skullcap is malleable in early childhood, so a unilateral head position during sleep can be a predisposing factor for cranial asymmetry. However, the effect of a unilateral sleeping position on facial asymmetry has rarely been studied in older children. It has been suspected that a habitual prone sleeping position can cause facial asymmetry due to increased asymmetric pressure on the facial area (44). In the present study, the linear regression models showed that children who slept mainly in the supine position at the age of 6 and 12 months had more symmetric faces later in childhood. While no conclusions about this issue can be drawn based on this sample, it might be speculated that prone-sleeping children vary their head position spontaneously during the night. Therefore, the pressure on their facial area may vary during sleeping.

Because torticollis is known to be an important predisposing factor for facial asymmetry (45–47), one focus in the present study was on analysing the effect of the neck range of motion. The majority of prior studies have set a 15 degrees difference in neck mobility as the cut-off point to define torticollis. Only a few children in this cohort exceeded the cut-off point. Therefore, the focus shifted to whether minor neck mobility imbalances could affect the development of facial asymmetry. Results from linear regression models showed that small head flexion or rotation imbalances in early childhood might predict facial asymmetry later in childhood, although the changes were small, and the results were partly contradictory.

No connections between occlusal and facial asymmetries were found at 6 years of age. However, unilateral crossbite is known to be an essential risk factor for the development of facial asymmetry. Primozic *et al*. (32,48). have studied that issue with analogous 3D soft tissue imaging methods. They found that children with a functional unilateral crossbite had more facial asymmetry than controls. In the lower facial area, asymmetry was observable in the deciduous dentition phase, and if the crossbite remained uncorrected, mid-facial asymmetry appeared during the early mixed dentition phase. In our study population, there were only three cases of crossbite at the age of 6 years. Presumably due to the small number of occlusal abnormalities, no association between asymmetric occlusion and facial asymmetry was found in the present study.

The role of breastfeeding in the development of facial musculature and occlusion has been studied in recent decades. There are reports showing that breastfeeding has a protective effect on the development of posterior crossbite (49), although opposite results have also been presented (50–52). In the present study, simple linear regression revealed a positive association between breastfeeding duration and facial symmetry.

Pacifier use and prolonged sucking habits are considered risk factors for occlusal asymmetries (50,51,53). In the present study, no connection was found between the duration of pacifier use and facial asymmetry. However, in those prior studies, the duration of pacifier use was remarkably longer. Currently, Finnish maternity clinics advise parents to discontinue pacifier use between 1 and 2 years of age. Indeed, in our study population, only one child was using a pacifier at the age of 3 years.

The method used in this study has been described in several prior studies (19,32,33,48,54,55). All facial symmetry studies have to deal with the difficulty of defining the reference plane (i.e. facial midline). As a complex 3D and asymmetrical structure, the facial area lacks an absolute midline. In 2D methods, the facial midline is usually defined by certain specific landmarks (38,56). 3D imaging enables the use of more sophisticated methods—mirroring and superimposing facial parts—to define the facial midline, which makes it possible to use all points on the facial surface (32,33,54). A limited facial area above the landmark subnasale was used in defining the midline by superimposition. In our opinion, superimposing the whole facial image might, in some cases with pronounced lower jaw asymmetry or incorrect lower jaw position, mask lower jaw asymmetry or erroneously increase reported asymmetry in upper parts of the face. Congruently, temporal areas were excluded from the defined area to minimize the effect of hair and other irregularities on the temporal area. Importantly, surface-based methods (average distance and symmetry percentage) involve scaling the facial images to the same size. Usually, the scaling reference is the average of all participants in each study. Scaling eliminates the error caused by growth and size differences in the study sample but does not allow comparing the exact numbers and percentages with other studies.

This study provides information about the development of facial asymmetry in early childhood. A slight improvement in facial symmetry parameters was observed in the whole study population, and some of the improvements reached statistical significance. However, as expected in a normal population, the changes cannot be considered clinically remarkable. Further, the size of this study population was limited. In the future, studies of 3D soft tissue imaging should focus on creating population-based normal values for facial asymmetry. Such values would help to distinguish abnormal development before it becomes clinically obvious.

## Conclusion

Facial asymmetry is detectable in early childhood. The lower face deviates slightly to the right, and the upper face to the left in relation to the facial midline. Facial symmetry tends to improve between 1 and 6 years of age, but the change in facial appearance is not considered clinically significant. Possible predictors of facial asymmetry at the age of 6 years include DP, sleeping position, and previously measured asymmetry.

## Supplementary Material

cjad012_suppl_Supplementary_Table_S1

## Data Availability

The data underlying this article cannot be shared publicly due to the privacy of individuals that participated in the study. The data will be shared on reasonable request to the corresponding author.
